# Slipping through the Cracks: The Taxonomic Impediment Conceals the Origin and Dispersal of *Haminoea japonica*, an Invasive Species with Impacts to Human Health

**DOI:** 10.1371/journal.pone.0077457

**Published:** 2013-10-03

**Authors:** Dieta Hanson, Samantha Cooke, Yayoi Hirano, Manuel A. E. Malaquias, Fabio Crocetta, Ángel Valdés

**Affiliations:** 1 Department of Biological Sciences, California State Polytechnic University, Pomona, California, United States of America; 2 Redpath Museum and Department of Biology, McGill University, Montréal, Québec, Canada; 3 Graduate School of Science, Chiba University, Chiba, Japan; 4 Phylogenetic Systematics and Evolution Research Group, University Museum of Bergen, Bergen, Norway; 5 Stazione Zoologica Anton Dohrn, Napoli, Italy; University of Waikato (National Institute of Water and Atmospheric Research), New Zealand

## Abstract

*Haminoea japonica* is a species of opisthobranch sea slug native to Japan and Korea. Non-native populations have spread unnoticed for decades due to difficulties in the taxonomy of *Haminoea* species. *Haminoea japonica* is associated with a schistosome parasite in San Francisco Bay, thus further spread could have consequence to human health and economies. Anecdotal evidence suggests that *H. japonica* has displaced native species of *Haminoea* in North America and Europe, becoming locally dominant in estuaries and coastal lagoons. In this paper we study the population genetics of native and non-native populations of *H. japonica* based on mt-DNA data including newly discovered populations in Italy and France. The conclusions of this study further corroborate a Northeastern Japan origin for the non-native populations and suggest possible independent introductions into North America and Europe. Additionally, the data obtained revealed possible secondary introductions within Japan. Although non-native populations have experienced severe genetic bottlenecks they have colonized different regions with a broad range of water temperatures and other environmental conditions. The environmental tolerance of this species, along with its ability to become dominant in invaded areas and its association with a schistosome parasite, suggest *H. japonica* could be a dangerous invasive species.

## Introduction

Existing gaps in taxonomic knowledge and the shortage of trained taxonomists to fill the need to identify living organisms are collectively known as the taxonomic impediment [1,2]. The taxonomic impediment has implications for invasion biology [3]. Because alien species can come from all over the world, proper identification of newly arrived species is a major challenge [3]. Misidentification of these species can have serious consequences. For example, early detection is critical to manage and control alien species [4], thus confusing newly arrived aliens with native species can delay early intervention and exacerbate the problem. When those alien species impact human health and economies or harm native populations [5,6], early detection and control become even more critical.

In this paper we study the spread of *Haminoea japonica*, an opisthobranch sea slug native to Japan and Korea with a non-native range including the west coast of North America, Spain, Italy and France [7,8]. Documentation of the spread of *H. japonica* has been hampered by the taxonomic impediment. For example, the first record of this species in North America was published as a new species, *Haminoea callidegenita* Gibson and Chia, 1989 [7]. Additionally, the first COI sequence of *H. japonica* published in GenBank (DQ238004) was misidentified as belonging to *Haminoea hydatis* (Linnaeus, 1758), a morphologically similar European species. Considering the current known range of *H. japonica* in Italy [9], it seems plausible or even likely that this species might have colonized large areas of the western Mediterranean and Adriatic Sea but remains undetected due to its external similarity to some native *Haminoea* species and the lack of trained biologist capable of proper identification of specimens. Most alarming is the fact that *H. japonica* appears to be an aggressive competitor that might have displaced native congeneric species in North America and Europe. For example, Hanson et al. [8] reported the complete replacement of a once-abundant population of *Haminoea vesicula* (Gould, 1855) in Boundary Bay (with up to 200 individuals per m^2^) by *H. japonica*. This is paralleled by a similar observation made in Laguna di *Sabaudia*, Italy in which *H. japonica* is now dominant [10]. The ecological and biodiversity implications of the spread of *H. japonica* remain poorly understood.

Previous research on the population genetics of *H. japonica* concluded that the non-native populations most likely originated in Northeastern Japan and dispersed via exports of commercial bivalves [8]. However, it was not possible to determine whether European populations were the result of a secondary invasion from North America, or an independent invasion from Japan. Although it was hypothesized that non-native populations of *H. japonica* appeared to be constrained by cold-water requirements, because they originated in colder regions of Japan (influenced by the Oyashio ocean current) [8], and the warm-water Mediterranean populations appeared to have gone extinct [11], recent records of established populations in two western Mediterranean coastal lagoons contradict this hypothesis [9].

**Figure 1:**
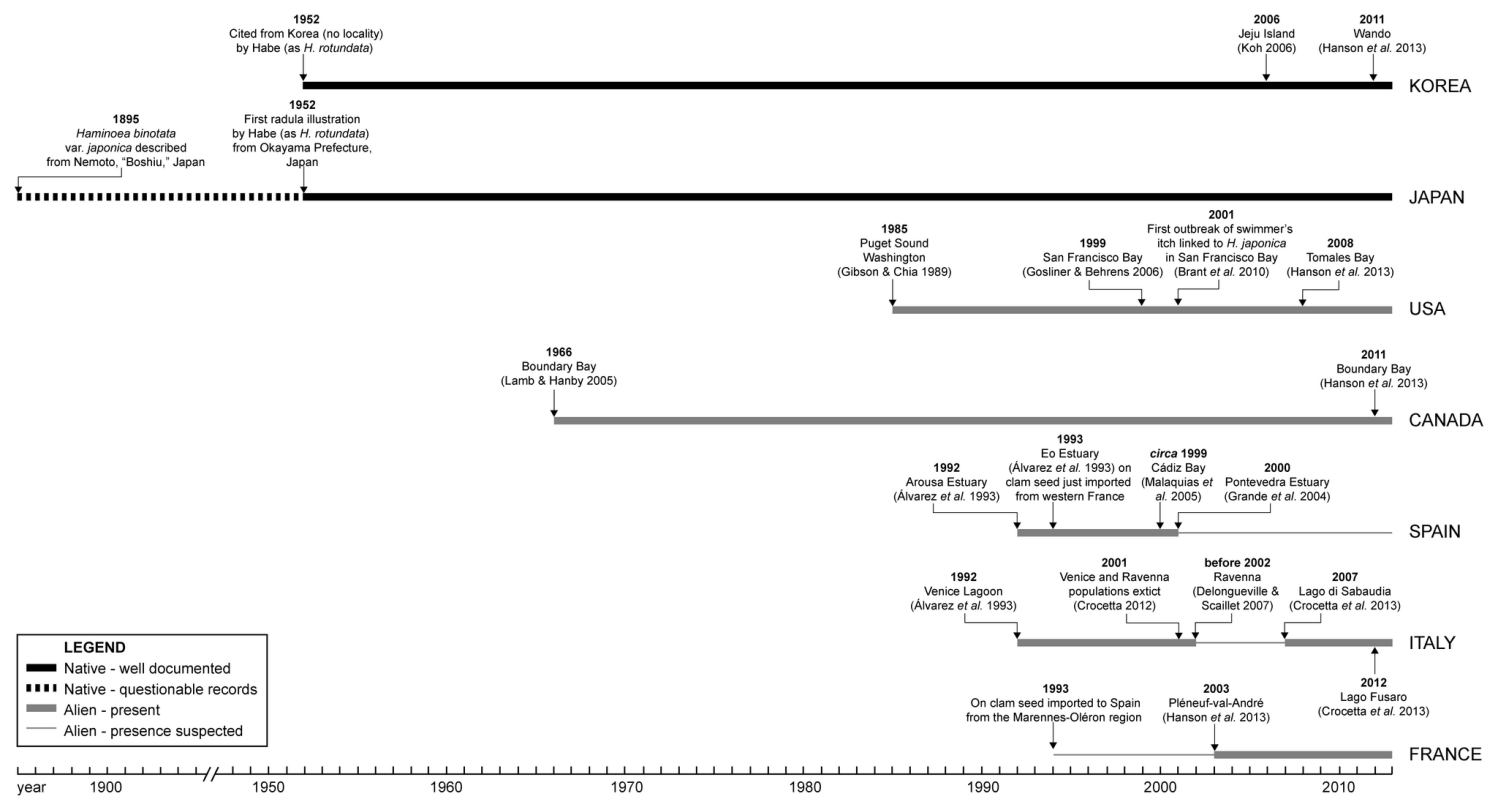
Timeline of the spread of *H. japonica* into North America and Europe.

The anthropogenic dispersal of *H. japonica* also has immediate implications for humans. In North America it serves as the only known intermediate host of a schistosome parasite that is responsible for annual cercarial dermatitis (swimmer’s itch) outbreaks in humans in San Francisco Bay, resulting in beach closures [12]. Cercarial dermatitis is now considered to be a global emerging disease, with increases in frequency attributed to the spread of invasive mollusks and global climate change [13]. Although cercarial dermatitis typically has mild symptoms, causing unpleasant irritation, it can have serious consequences for tourism-based economies if it results in prolonged and/or recurrent beach closures. Comprehensive knowledge of the schistosome life cycle, including detailed knowledge of its hosts, is vital to understand and manage risks to human health and economies. However, several important factors in the *H. japonica*-schistosome association are still unknown. The schistosome species responsible for the outbreaks in San Francisco Bay has not been identified, as it does not match any available DNA sequences in GenBank, and does not bear a morphological resemblance to any other schistosomes known from the area [12]. This implies two possible origins for the parasite. First, it could be a native species that uses a native snail as a host, but switched hosts once *H. japonica* was introduced. This is unlikely, since before *H. japonica* introductions (Figure 1), reports of cercarial dermatitis were rare, and tests for infection of this schistosome in snails native to San Francisco Bay were negative [12]. Second, the parasite could have been introduced to San Francisco Bay, either with *H. japonica* from Japan, or from a different location via migratory birds, which carry the adult form of the schistosomes. Regardless of the origin of the parasite, further spread of schistosome-carrying *H. japonica* could bring outbreaks of swimmer’s itch into adjacent areas such as Southern California, whose economies heavily rely on recreational water activities.

In this paper, we study four previously unknown populations of *H. japonica* in the Mediterranean and a number of new specimens collected from the native and non-native ranges. To provide additional evidence for the Asian-Pacific origin of *H. japonica* we constructed a multigene phylogeny of *Haminoea* including a combination of Atlantic, eastern Pacific and Indo-Pacific taxa. Finally, we tested populations of *H. japonica* in the native range for schistosome parasites to determine whether the parasite could have a Japanese origin. The overall goals of this paper are: 1) To provide additional evidence on the origin, current range, dispersal pathway and invasive potential of *H. japonica*; 2) To raise awareness of what could become a dangerous invasive species to the ecology of estuaries in the Mediterranean and North America, with potential impacts to human health and therefore to the economies of these coastal regions; 3) To provide a large sample of the genetic variation of *H. japonica* to facilitate molecular identification of newly discovered populations.

## Materials and Methods

### Population genetics

To study the population genetics of *Haminoea japonica* in the native and non-native ranges, DNA was extracted, amplified, and sequenced for the mitochondrial gene cytochrome *c* oxidase subunit I (COI) as described in Hanson et al. [8]. Thirty-four individuals from Japan and eleven individuals from Europe were added to the samples studied by Hanson et al. [8] and Crocetta et al. [9]. This resulted in a total sample size of 142 individuals, spanning the entire known range of the species. Sequences were assembled and edited using the software Geneious Pro 4.8.3 [14]. Geneious Pro 4.8.3 was also used to extract the consensus sequences and to align them using the default parameters. A haplotype network was constructed using TCS 1.21 [15] with a 95% connection limit. To assess the potential for missed haplotypes due to sample size in both the native (Japan and Korea) and non-native (North America and Europe) range, rarefaction analyses were run in EstimateS 8+ [16] with each location considered a single sample, even though collections may have been made at each location in more than one occasion. North American and European samples were run together under a single analysis since the low number of samples from Europe would make the identification of the rarefaction curve asymptote impossible. Chao2 means and 95% lower and upper confidence intervals were estimated.

Based on geographic distribution, five groups (Northeastern Japan, Southern Japan, Sea of Japan, North America, and Europe) and 27 populations (see Table 1) were tested for genetic structure using the analysis of molecular variance (AMOVA) implemented in Arlequin 3.5 [17]. Three AMOVA analyses were run for all localities, native range localities and non-native range localities. Significance of the AMOVAs was tested using 16,000 permutations of individuals between groups. Arlequin 3.5 was also used to calculate F_ST_ values as a measure of pairwise differences between all population groups (Table 1) as well as the nucleotide diversity (π) and haplotype diversity (h) of each population. The significance of the pairwise F_ST_ value was estimated by performing 16,000 permutations. The F_ST_ analysis was conducted with population groups rather than populations because of two reasons, the large number of populations would result in lower confidence values after Bonferroni’s correction and the presence of many populations with one or a small number of haplotypes would render the results uninformative or difficult to interpret.

**Table 1:** List of populations studied in the population genetics analyses including haplogroup codes, haplotype IDs, and COI GenBank accession numbers for all specimens examined.

**Population**	**Number of specimens**	**Haplogroup**	**Haplotype IDs**	**GenBank Accession Numbers**
Amakusa, Japan	4	A, C	H1, H2, H3, H4	JN830648-JN830651
Atsumi Peninsula, Japan	3	B	H16, H30	KF572952-KF572954
Boundary Bay, Canada	2	H	H23	JN830721-JN830722
Hachinohe, Japan	4	F	H5, H6, H7	JN830652-JN830655
Hakodate, Japan	8	F	H5, H7, H8, H9	JN830656-JN830663
Hiroshima, Japan	2	B, C	H27, H28	JQ693573-JQ693574
Hitachinaka, Japan	9	G, H	H20, H21, H22	JN830692-JN830700
Lago di *Sabaudia*, Italy	4	H	H20, H25	JX679602-JX679605
Lago Fusaro, Italy	5	H	H25	JX679598-JX679601
Laguna di Orbetello, Italy	1	H	H23	KF572955
Latina, Italy	2	H	H20	KF572956-KF572957
Le Barcarès, France	3	H	H20	KF615822-KF615824
Mangokuura, Japan	16	B, E, H	H19, H20, H23, H27, H29, H31	JQ693575, KF572970-KF572984
Matsushima Bay, Japan	12	H	H20, H23, H32	KF572958-KF572969
Minamisatsuma, Japan	4	A	H33, H34, H35	KF572985-KF572988
Pléneuf-val-André, France	2	H	H20	JN830727-JN830728
Po Delta (Piallassa Baiona, Sacca di Goro), Italy	5	H	H23	KF572989-KF572993
Pontevedra, Spain	4	H	H20, H25	JN830729-JN830732
Sado Island, Japan	4	E, F	H5, H10, H11	JN830664-JN830667
Sagami Bay, Japan	2	D	H13	JN830669-JN830670
San Francisco Bay, California	12	H	H23	JN830703-JN830714
San Juan Islands, Washington	9	H	H20, H23	JN830723-JN830725, JN830718-JN830722
Souma, Japan	1	H	H26	JQ693572
Tokyo Bay	12	B, C, D, E	H13, H14, H15, H16, H17	JN830671-JN830682
Tomales Bay, California	3	H	H20, H23, H24	JN830715-JN830717
Uchiura Bay, Japan	9	B, C	H16, H18, H19	JN830683-JN830691
Wando, South Korea	1	F	H12	JN830668

### Phylogenetic analysis

To gain insight into the geographic origins of *H. japonica*, the species was placed within a larger phylogeny of the genus *Haminoea*. For this part of the study DNA was extracted, amplified, and sequenced for the mitochondrial gene cytochrome c oxidase subunit I (COI) and nuclear gene 28S rRNA, as described by Malaquias et al. [18]. Sequences were verified by both forward and reverse comparisons and assembled and edited using Sequencher 5.01 (Gene Codes Corp.). All sequences were deposited in GenBank (Table 2). Sequences were aligned using Clustal_X [19] with a slow-accurate method and default settings. The alignments were further optimized by eye using MacClade 4.06 [20]. The best-fit models of evolution (TVM + I + G for COI and GTR+ I + G for 28S rRNA) were chosen using the Akaike information criterion [21] implemented in ModelTest 3.6 [22]. Aligned sequences were trimmed to exclude ambiguous reads at each end and gap-rich regions where homology could not be confidently assessed. A total of 522 bp of COI and 720 bp of 28S rRNA remained for use in phylogenetic analyses comprising 44 sequences. A combined analysis of the two genes (total 1242 bp) was conducted using Bayesian inference analysis in MrBayes 3.1.2b with default priors [23,24] for 1.5 x 10^6^ generations, with a sampling frequency of 100 and two separate runs to ensure that independent analyses were converging on the same tree. Convergence of runs was diagnosed using the program Tracer 1.4 [25]. For each analysis the first 3750 trees were discarded (‘burn-in’ period). Robustness of each node was assessed using Bayesian posterior probabilities calculated by MrBayes 3.1.2b. Two members of the family Bullidae (*Bulla occidentalis* A. Adams, 1850 and *Bulla striata* Bruguière, 1792) were chosen as outgroup taxa.

**Table 2:** List of specimens used for phylogenetic analysis, with sampling localities, GenBank accession numbers and location of voucher specimens.

**Taxon**	**Locality**	**Voucher Number**	**GenBank Accession No.**	
			**COI**	**28S**
*Haminoea* sp.3 (C26)	Philippines	MNHN 42261	KF615810	KF615809
*Haminoea* sp.2 (C3)	Indonesia	NHMUK 20050660	DQ974673	DQ927230
*Haminoea* sp.2 (93)	East Timor	NHMUK 20060109	KF615835	KF615808
*Haminoea* sp.1 (C37)	Philippines	MNHN 42265	KF615821	KF615788
*H. japonica* (C52)	Mediterranean Sea	NHMUK 20070029	KF615824	KF615786
*H. japonica* (149)	Mediterranean Sea	NHMUK 20070065	KF615823	KF615787
*H. japonica* (164)	Mediterranean Sea	NHMUK 20070028	KF615822	KF615785
*H. natalensis* (68)	United Arab Emirates	NHMUK 20060104	KF615826	KF615783
*H. natalensis* (153)	South Africa	NHMUK 20070186	KF615825	KF615784
*H. cymbalum* (C28)	Philippines	MNHN 42249	DQ974675	DQ927232
*H. cymbalum* (16)	Indonesia	NHMUK 20030302	KF615842	KF615807
*H. ovalis* (C34)	Philippines	MNHN 42252	DQ974677	DQ927234
*H. navicula* (C51)	UK	NHMUK 20060324	DQ974676	DQ927233
*H. navicula* (129)	Portugal	NHMUK 20070018	KF615838	KF615804
*H. navicula* (130)	Portugal	NHMUK 20070020	KF615837	KF615803
*H. navicula* (131)	Portugal	NHMUK 20070020	KF615839	KF615805
*H. navicula* (147)	UK	NHMUK 20070021	KF615836	KF615806
*H*, cf. *hydatis* (C53)	Mediterranean Sea	NHMUK 20060326	DQ974674	DQ927231
*H*, cf. *hydatis* (166)	Mediterranean Sea	NHMUK 20060326	KF615841	KF615802
*H*, cf. *fusari* (167)	Italy	NHMUK 20070177	KF615840	KF615801
*Haminoea* sp.4 (152)	Florida, USA	NHMUK 20070180	KF615829	KF615797
*Haminoea* sp.4 (188)	Florida, USA	NHMUK 20070448	KF615828	KF615798
*Haminoea* sp.4 (175)	Florida, USA	NHMUK 20070318	KF615827	KF615800
*Haminoea* sp.4 (189)	Florida, USA	NHMUK 20070448	KF615831	KF615799
*Haminoea* sp.4 (190)	Florida, USA	NHMUK 20070603/1	KF615832	KF615795
*Haminoea* sp.4 (191)	Florida, USA	NHMUK 20070603/2	KF615830	KF615796
*Haminoea* sp.5 (161)	Mexico	NHMUK 20070090	KF615833	KF615793
*Haminoea* sp.5 (154)	Mexico	NHMUK 20070175	KF615834	KF615794
*H. orteai* (197)	Azores, Portugal	NHMUK 20070458	KF615844	KF615791
*H. orteai* (198)	Azores, Portugal	NHMUK 20070458	KF615845	KF615790
*H. orteai* (48)	Canary Is, Spain	NHMUK 20030836	KF615846	KF615792
*H. vesicula* (202)	California, USA	CASIZ 97502	KF615843	KF615789
*H. alfredensis* (174)	South Africa	NHMUK 20070314	KF615816	KF615774
*H. alfredensis* (182)	South Africa	NHMUK 20070315	KF615815	KF615775
*H. alfredensis* (183)	South Africa	NHMUK 20070315	KF615814	KF615773
*H. antillarum* (176)	Florida, USA	NHMUK 20070316	KF615817	KF615778
*H. antillarum* (157)	Mexico	NHMUK 20070091	KF615819	KF615782
*H. antillarum* (158)	Mexico	NHMUK 20070094	KF615811	KF615779
*H. antillarum* (159)	Mexico	NHMUK 20070092	KF615818	KF615780
*H. antillarum* (160)	Mexico	NHMUK 20070093	KF615820	KF615781
*H. orbignyana* (1)	Portugal	NHMUK 20030296	KF615813	KF615776
*H. orbignyana* (148)	Portugal	NHMUK 20030296	KF615812	KF615777
*Bulla striata* (79)	Senegal	NHMUK 20030784/3	DQ986566	DQ986693
*Bulla occidentalis* (139)	Panama	BMNH 20060118	DQ974658	DQ927209

Abbreviations: CASIZ, California Academy of Sciences (Invertebrate Zoology), San Francisco; MNHN, Museum National d’Histoire Naturelle, Paris; NHMUK The Natural History Museum, London

### Assessment for parasite infection

In order to test for a possible Japanese origin of the parasites associated with *H. japonica* in San Francisco Bay, 108 individuals collected in Uchiura Bay, Japan and 30 individuals collected in Matsushima Bay, Japan were assessed for schistosome parasite infections. No permits are necessary to study and/or collect *Haminoea japonica* on the two sites. A permit from the local fishermen’s association is required to collect commercially valuable species, but *H. japonica* is not commercially valuable or an endangered species.

After collection, specimens were kept in containers filled with seawater and exposed to bright, direct sunlight for 1-2 hours. The specimens were then removed and the water in the container was examined for cercaria under a stereomicroscope or with the naked eye. Additionally, a subset of 72 specimens from the 108 specimens collected in Uchiura Bay, were dissected and examined for schistosome infection of the digestive gland.

## Results

### Phylogeny of *Haminoea*

**Figure 2:**
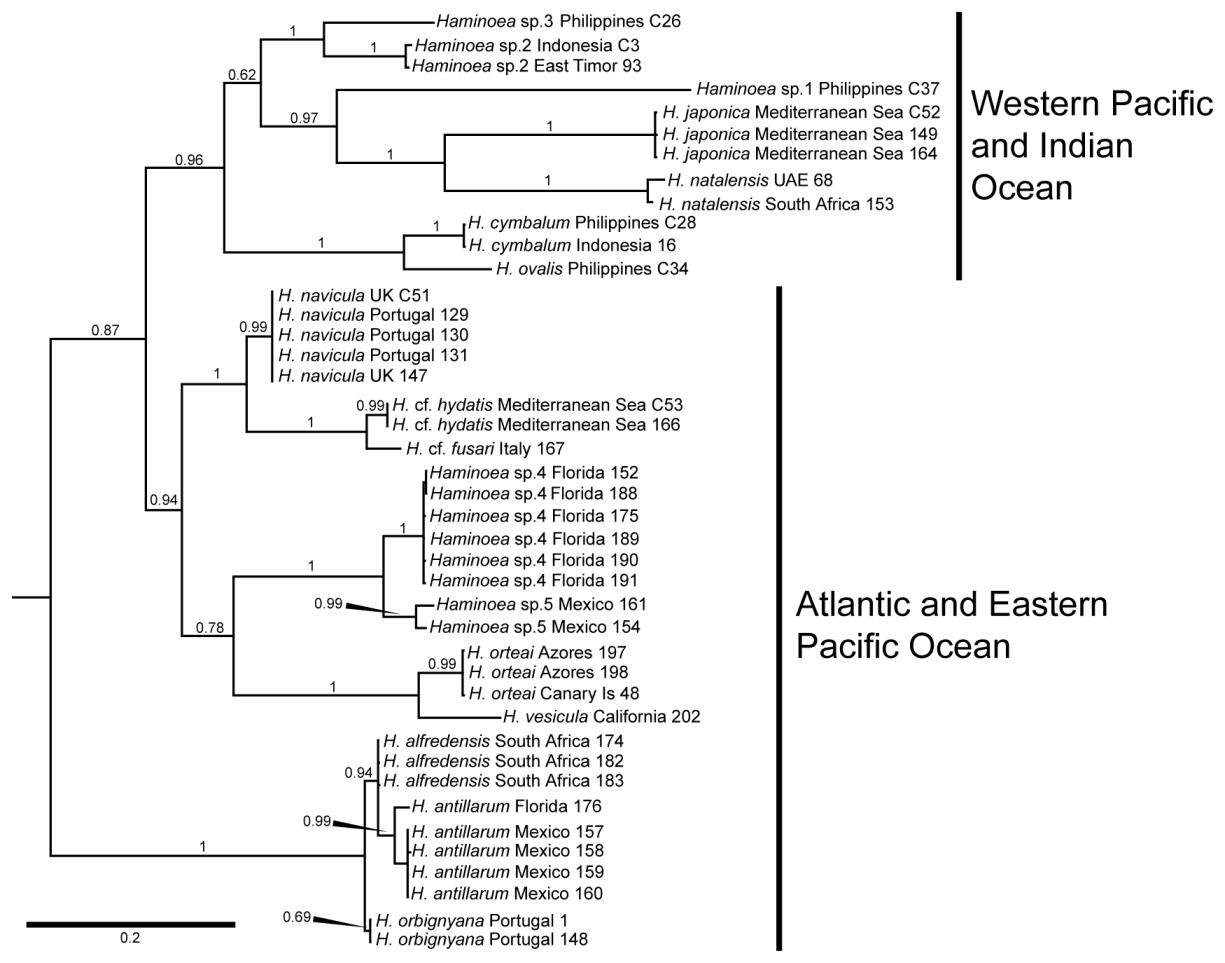
Bayesian molecular phylogeny produced from the combined mitochondrial COI and nuclear 28S rRNA genes. Numbers above branches are posterior probabilities. Outgroup taxa removed for clarity.

The phylogeny of *Haminoea* shows *H. japonica* as monophyletic and sister to *Haminoea natalensis* (Krauss, 1848), a species described from the Indian Ocean coast of South Africa. These two species are placed within a highly supported (posterior probability = 0.96) clade of Southeast Asian species (Figure 2). Sister to this is a clade containing seven species, six of which are Atlantic or Mediterranean in origin, and one Eastern Pacific (*Haminoea vesicula*).

### Population genetics

The AMOVA results (Table 3) revealed that when all populations are analyzed together the majority of genetic variation (50.9%) occurred within populations, whereas the variation among groups was 36.74%. The AMOVA run based on native range populations only gave similar results, with most of the variation (52.87%) within populations and only 32.7% among groups. However, the AMOVA run for non-native populations only, calculated much higher genetic variation among populations within groups (46.23%) and very little among groups (15.43%). The F_ST_ analysis results (Table 4) confirmed that all population groups examined are genetically distinct from one another with the exception of Northeastern Japan, which is not significantly different from North America and Europe.

**Table 3:** Results of the AMOVA analyses for all populations, populations in the native range and populations in the non-native range.

**Analysis**	**Partitioning**	**d.f.**	**Sum of squares**	**Variance component**	**% variation**	**F-statistics**
All	Among groups	4	334.700	2.62927	36.74	F_CT_=0.36742*
	Among populations within groups	22	171.404	0.88437	12.36	F_SC_=0.19537*
	Within populations	115	418.875	3.64239	50.90	F_ST_=0.491*
	Total	141	924.979	7.15604		
Native	Among groups	2	228.837	3.35116	32.70	F_CT_=0.32702*
	Among populations within groups	12	161.142	1.47896	14.43	F_SC_=0.21445*
	Within populations	76	411.736	5.41758	52.87	F_ST_=0.47134*
	Total	90	801.714	10.2477		
Non-native	Among groups	1	3.461	0.07369	15.43	F_CT_=0.15434
	Among populations within groups	10	10.263	0.22070	46.23	F_SC_=0.54663*
	Within populations	39	7.139	0.18305	38.34	F_ST_=0.6166*
	Total	50	20.863	0.47744		

Significant values marked with an asterisk.

**Table 4:** Matrix of the population group comparisons results, with F_ST_ values (lower triangular) and associated *p* values (upper triangular).

	NE Japan	S Japan	Sea of Japan	N America	Europe
NE Japan	-	0.0000*	0.0000*	0.005	0.0479
S Japan	**0.24897**	-	0.0000*	0.0000*	0.0000*
Sea of Japan	**0.75648**	**0.29864**	-	0.0000*	0.0000*
N America	0.09066	**0.29751**	**0.93147**	-	0.0001*
Europe	0.05459	**0.27706**	**0.91303**	**0.25547**	-

After Bonferroni correction (10 comparisons) significant values are *p* < 0.005. Significantly distinct groups in bold, significant *p* values marked with an asterisk.

**Figure 3:**
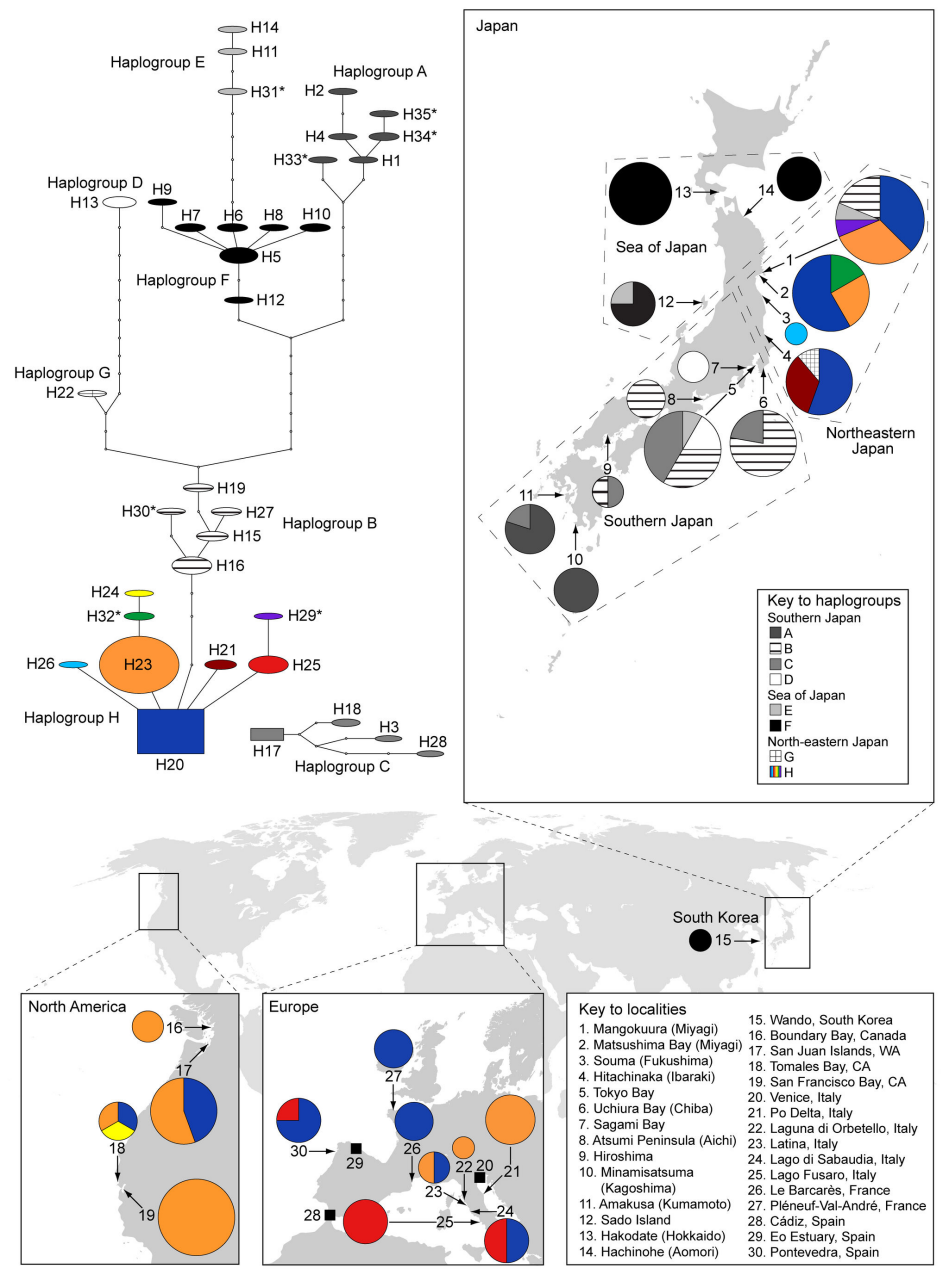
Haplotype network and distribution map of records of *H. japonica* in Japan, Korea, Europe and North America. Numbers in the map indicate the localities listed in the key to localities. Numbers in the haplotype network indicate haplotype number as in Table 1. Haplotypes marked with an asterisk have been newly found in this study.

**Figure 4:**
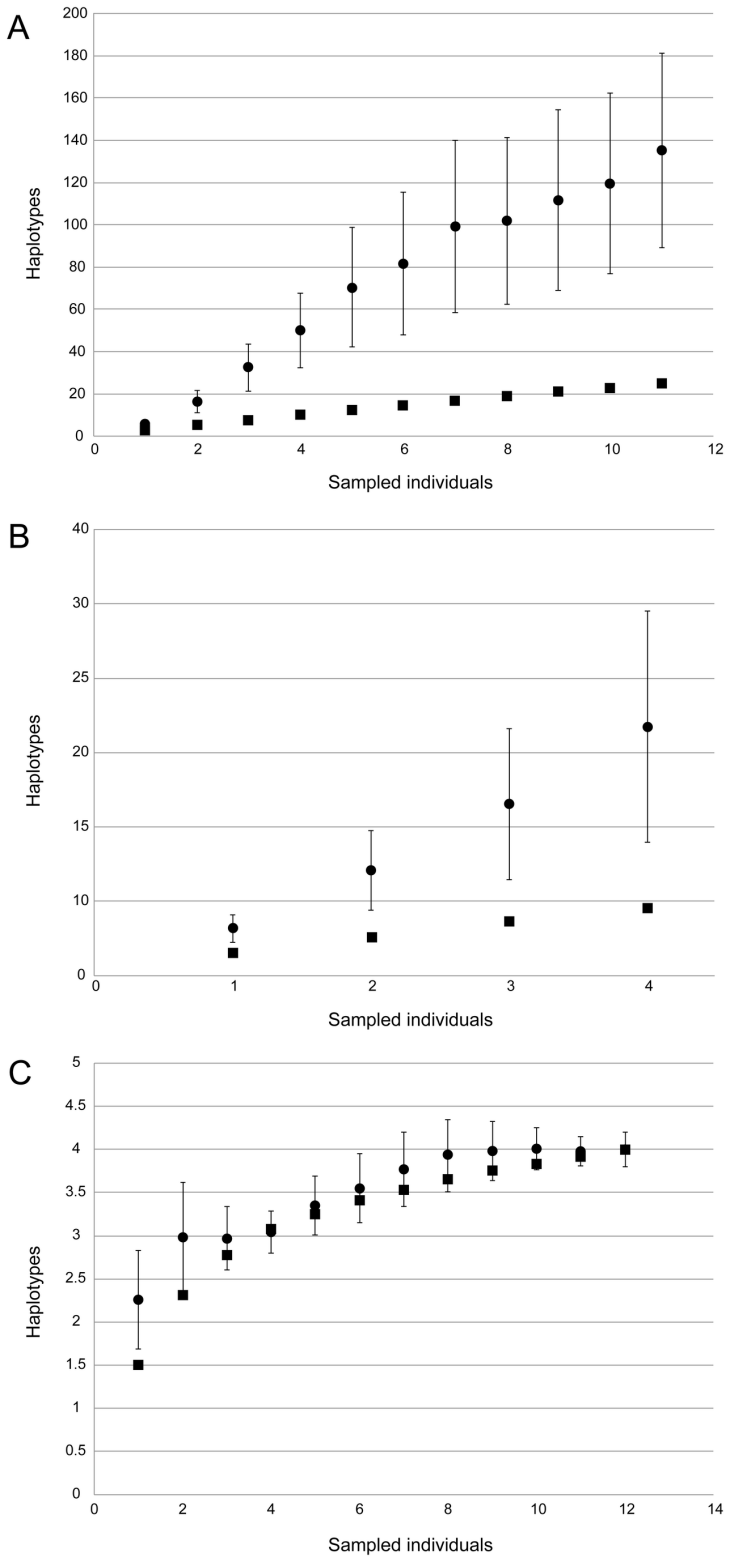
Rarefaction analyses of COI haplotype diversity with sampling effort in *H. japonica*. Black circles = Chao2 mean, Black squares = Sobs. A. Native range excluding Northeastern Japan. B. Northeastern Japan. C. Non-native range.

Thirty-three haplotypes were identified in the native range, and four in the non-native range (Figure 3). Two of the four non-native haplotypes were found in Japan, whereas two (H24 – from a single individual collected from Tomales Bay and H25 – widespread in Spain and Italy) were not detected in the native range. Rarefaction analysis of the non-native range haplotypes predicted that four haplotypes should exist in the non-native range (Chao2 mean and 95% CI lower bound), with a maximum of 4.46 (95% CI upper bound). This suggests that all the haplotypes present in the non-native range have been recovered, although further sampling may reveal one additional haplotype. Because both the sample data and the Chao2 estimate curves reached an asymptote (Figure 4C), these results can be considered reliable. The same analysis run for the northeastern Japan populations suggested that a minimum of 12.59 (lower 95% CI) and a maximum of 176.22 (upper 95% CI) with a Chao2 mean of 33.5 haplotypes should be present. Although this maximum is not as reliable as that for the non-native range analysis, since the rarefaction curves did not reach an asymptote (Figure 4B), the minimum 12.59 estimate reveals that a substantial sampling effort is necessary to completely recover the genetic diversity existing in this region. Finally, a rarefaction analysis for the rest of the native range revealed that between 51.45-484.5 (135.25 Chao2 mean) haplotypes should be present in the rest of Japan and Korea, meaning at least 31 more haplotypes could be detected with further sampling effort (Figure 4A).

### Parasite infection rates in Japan

None of the individuals examined for cercariae, either through shedding or dissection, were found to be infected with schistosome parasites.

## Discussion

### Origin and population structure of the non-native populations of *H. japonica*

The phylogenetic analysis of *Haminoea* suggests an Indo-Pacific origin of *H. japonica* [7] as this species is nested in a clade containing only Indo-West Pacific species (Figure 2). However, the placement of *H. japonica* as sister of *H. natalensis* is probably an artifact of the limited taxon sampling within the Indo-pacific species examined. Additionally, the F_ST_ analysis results (Table 4) confirmed the population differentiation and structure revealed with a smaller sample size [8]. This is consistent with the hypothesis of a northeastern Japan origin for the non-native populations.

The results of the AMOVA analyses are indicative of substantial gene flow between populations in the native range resulting in most of the genetic variation occurring within populations (52.87%) and very little among populations (14.43%). However, there is substantial variation among groups (32.70%), suggesting a certain degree of isolation between the Sea of Japan, Southern Japan and Northeastern Japan populations of *H. japonica*.

As suggested below, the movement of bivalves between regions in Japan may have resulted in the introduction of southern haplotypes of *H. japonica* to Mangokuura in Northeastern Japan. Introductions of *H. japonica* into the non-native range have been associated with commercial imports of bivalves, particularly oysters and clams [7,8,11], but this new evidence suggests the same vector have resulted in movements of *H. japonica* within the native range, but between distinct populations. Such movements may have also brought haplotypes of diverse origins to Tokyo Bay, resulting in lower percentages of variation among groups than within populations. However, considering the high intensity of historic and recent transport of bivalves between regions of Japan and Korea [26], the impact of these within-Japan introductions in the present genetic structure of *H. japonica* is surprisingly low, as revealed by the relatively high percentage of variation among groups. This suggests that introductions of *H. japonica* are probably rare.

The genetic structure in the non-native range is very different, with very little variation among groups (15.43%) and high percentages of differentiation among populations and within populations (46.23% and 38.34% respectively). The genetic uniformity between Europe and North America suggest that these populations are the result of either a single or a small number of introduction events, whereas the high percentage of variation among populations is likely the result of founder effects.

### Sample size and rarefaction curves

The rarefaction analysis confirms that this study has recovered all or most of the COI haplotypes found in the non-native range of *H. japonica* (only one potentially remains unrecovered). However, a number of haplotypes from the native range remain undetected by our sampling effort. This would explain the fact that haplotypes H24 and H25 are missing from our sample in the native range. The present analysis confirms that the reduced genetic diversity in the non-native range is not an artifact of a small sample size, but it is indicative of severe population bottleneck, which is consistent with a relatively recent introduction from a single or a small number of sources. Further evidence of population bottleneck in the non-native populations is provided by the low levels of both nucleotide and haplotype diversity in North America and Europe (Table 5).

**Table 5:** Nucleotide (π) and haplotype (h) diversity for all populations examined except those with only one individual (Wando, Laguna di Orbetello, Souma).

**Population**	**Nucleotide diversity (π)**	**Haplotype diversity (*h*)**
Boundary Bay	0.00000	0.0000
San Francisco Bay	0.00000	0.0000
San Juan Islands	0.00084	0.5556
Tomales Bay	0.00304	1.0000
**North America Mean**	0.00097	0.3889
Lago di *Sabaudia*	0.00076	0.5000
Lago Fusaro	0.00000	0.0000
Latina	0.00000	0.0000
Le Barcarès	0.001013	0.6667
Pléneuf-Val-André	0.00000	0.0000
Po Delta	0.00000	0.0000
Pontevedra	0.00152	0.5000
**Europe Mean**	0.003293	0.2381
**Non-native Mean**	0.000652	0.2929
Amakusa	0.03926	1.0000
Atsumi Peninsula	0.00202	0.6667
Hachinohe	0.00177	0.8333
Hakodate	0.00152	0.6429
Hiroshima	0.00745	1.0000
Hitachinaka	0.00388	0.6389
Mangokuura	0.01167	0.7667
Matsushima Bay	0.00108	0.6212
Minamisatsuma	0.00455	0.8333
Sado Island	0.00633	0.8333
Sagami Bay	0.00000	0.0000
Tokyo Bay	0.04932	0.7879
Uchiura Bay	0.03073	0.6667
**Native Mean**	0.012275	0.7146

### Invasion pathway

With the available evidence it appears that the introductions of *H. japonica* in North America and Europe are independent events. The presence of one unique haplotype in each North American and European populations (H24 and H25 respectively) suggest independent introductions. If the European populations were the result of a secondary introduction from North America it would be difficult to explain the presence of the common European haplotype H25, which has not been detected in North America. Although these two haplotypes (H24 and H25) have not been detected in Japanese samples, suggesting they are rare in the native range, very similar haplotypes were recovered in Northeastern Japan (H29 and H32). The European haplotype H25 is common (frequency 36%) and widespread (found in Spain and Italy), this is likely the result of founder effect. On the contrary the North American haplotype (H24) is rare (frequency 4%).

### Secondary invasions within Japan

The presence in Tokyo Bay of several haplotypes commonly found in other regions of Japan was suggested to be the result of introductions of slugs from other populations [8]. In this paper for the first time we report the presence of haplotypes commonly found in Southern Japan in Northern Japan samples, but only in the coastal lagoon of Mangokuura in Miyagi Prefecture. Mangokuura is a major hub for bivalve culture and oyster spat exports and during the 1980-1990s received imports of oyster spat from a few locations in the Seto Inland Sea (Southern Japan) including Hiroshima and Okayama (Kenji Okoshi, pers. comm.). It is a distinct possibility that southern Japanese haplotypes of *H. japonica* could have been introduced into Mangokuura as a consequence of these activities. These secondary introductions are difficult to detect in the absence of molecular data and could be problematic, as alleles from non-native populations can spread through hybridization potentially reducing fitness of native populations.

### Potential for further spread

Previous work suggested that cold winter ocean temperatures could limit the potential range of non-native populations of *H. japonica* [8]. This hypothesis was based on two observations: 1) The non-native populations originated in a region influenced by the Oyashio cold current; 2) All known non-native records by the time of the publication of the paper were from regions with relatively cold water temperatures such as northern California and Washington, USA, the Atlantic coasts of Spain and France, and the northern Adriatic Sea. This was corroborated by the assumption that the Mediterranean populations have become extinct [11] due to absence of recent records even in well-sampled areas such as the northern Adriatic Sea lagoons. However, evidence of established populations of *H. japonica* in coastal lagoons along the western coast of Italy [9] suggested that the potential spread of this species could be much greater. In the present paper we report *H. japonica* for the first time in the Mediterranean coast of France as well as in three new localities in Italy. It appears that *H. japonica* is now well established in the western Mediterranean and central Adriatic and that milder winter temperatures do not appear to have prevented further spread. At this point all coastal lagoons in the Mediterranean as well as in Southern California should be considered vulnerable to invasion by *H. japonica*. It is possible that the range of *H. japonica* is already much larger than currently known, but it remains undetected due to difficulties in identification. The main external diagnostic characteristic of *H. japonica* is the presence of a deep notch in the cephalic shield [7], which is also present in the Atlantic and Mediterranean species *H. hydatis* [27], making these two species difficult to distinguish in the absence of molecular or anatomical data. These morphological similarities hamper proper identification and early detection of *H. japonica*.

### Potential ecological consequences of the invasion of *H. japonica*

Although based on anecdotal evidence [8], *H. japonica* seems to have displaced the Pacific Northwest species *H. vesicula* in British Columbia, suggesting it could be an aggressive invader. This is paralleled by a similar observation held in Laguna di *Sabaudia* [10] where *H. japonica* has become a dominant organism. The spread of *H. japonica* could have severe consequences for the biodiversity of *Haminoea* in the Mediterranean, where a number of species with restricted ranges occur in coastal lagoons. For example, *Haminoea fusari* is only known from Lago Fusaro, Italy, a coastal lagoon currently occupied by an established population of *H. japonica* [9]. Other species such as *Haminoea hydatis*, *H. navicula*, *H. orbignyana*, *H. orteai* and *H. templadoi* have been reported in southern European coastal lagoons, often in association with green algae (*Ulva*) and sea grasses [27,28,29,30,31] and are potentially vulnerable to the spread of *H. japonica*, which occupies a similar niche. Neither the low genetic diversity of non-native populations of *H. japonica*, nor their cold-water origin, seem to have hindered the rapid spread of this species in warmer Mediterranean waters and costal lagoons. Although *H. japonica* is currently considered a non-native species, it is possible that it has already become an aggressive invasive species and efforts to control it could become increasingly difficult.

### Potential human health consequences of the invasion of *H. japonica*

Examination of specimens in Japan did not reveal the presence of schistosome parasites. These results do not reveal anything new about the origin of the cercarial parasite associated with *H. japonica* in San Francisco Bay, but fail to provide evidence of a Japanese origin. The apparent absence of parasites associated with *H. japonica* in Washington State (USA), Canada and Europe seems to confirm that the San Francisco case is unique. It is thus likely that the parasite has traveled independently from *H. japonica* into San Francisco Bay [12] resulting in a serendipitous association. Regardless of the origin of the cercarial parasite, this association makes northern California populations of *H. japonica* susceptible to spread the parasite to nearby locations. Since water temperatures did not appear to prevent the spread of *H. japonica* in the Mediterranean, it is unlikely to do so in California. Thus, southern Californian estuaries, some of which are heavily dependent on water-related tourist activities, may be vulnerable to invasion by both the slug and the parasite, potentially resulting in economic loss.
